# Benchmarking Chemical Hydrolysis and Bacterial Biosynthesis Pathways for Nanocellulose: A Sustainability-Focused Comparative Framework

**DOI:** 10.3390/polym18030342

**Published:** 2026-01-28

**Authors:** Luis C. Murillo-Araya, Melissa Camacho-Elizondo, Diego Batista Meneses, José Roberto Vega-Baudrit, Mary Lopretti, Nicole Lecot, Gabriela Montes de Oca-Vásquez

**Affiliations:** 1 School of Mechatronics Engineering, Invenio University, Cañas 50601, Costa Rica; luisca232@gmail.com; 2 National Nanotechnology Laboratory (LANOTEC), National Center for High Technology (CeNAT), CONARE, Pavas 10109, Costa Rica; kcamachoe@utn.ac.cr (M.C.-E.); dbatista@cenat.ac.cr (D.B.M.); 3 Center for Sustainable Development Studies, Universidad Técnica Nacional, Alajuela 1902-4050, Costa Rica; 4 Escuela de Química, Universidad Nacional, Heredia 40101, Costa Rica; 5 Laboratory of Técnicas Nucleares Aplicadas a Bioquímica y Biotecnología, Nuclear Research Center, Faculty of Sciences, Mataojo 20 55, Montevideo CP 11400, Uruguay; mlopretti@gmail.com (M.L.); nlecot@fcien.edu.uy (N.L.)

**Keywords:** nanocellulose, pineapple agro-waste, acid hydrolysis, bacterial biosynthesis, *Rhizobium leguminosarum*, process optimization, decision matrix, sustainability assessment

## Abstract

This study benchmarks two nanocellulose (NC) production architectures: sulfuric-acid hydrolysis of pineapple peel biomass to obtain hydrolyzed nanocellulose (HNC) and microbial biosynthesis of bacterial nanocellulose (BNC) by *Rhizobium leguminosarum* biovar *trifolii* in defined media. HNC and BNC were characterized by SEM, FTIR, AFM, and ζ-potential, and the routes were compared using a sustainability-focused multicriteria framework. The Visual Integration of Multicriteria Evaluation (VIME) (radar chart + weighted decision matrix) yielded a higher overall score for BNC (66) than HNC (51), driven primarily by lower downstream washing/neutralization water demand (~0.3 L vs. ~14 L per batch), fewer purification stages (~2 vs. ~5), and lower waste hazard. In contrast, HNC performed better in calendar time (~7 vs. ~18 days). AFM revealed route-dependent morphologies: BNC formed a homogeneous nanofiber network (37 ± 9 nm), while HNC formed heterogeneous lamellar fragments (70 ± 12 nm). Route-specific yields were 3.15% (*w*/*w*, dry biomass basis) for HNC and 1.065 g/L (culture-volume basis) for BNC. Although a full ISO-compliant Life Cycle Assessment (LCA) and Techno-Economic Analysis (TEA) are beyond the scope of this laboratory-scale study, the defined system boundaries and reported process inventories provide an LCA/TEA-ready template for future mass- and cost-balanced comparisons.

## 1. Introduction

Cellulose is the most abundant biopolymer on Earth and one of the main components of the plant cell walls [1,2,3,4]. It is a linear polymer of β-(1→4)-linked glucose units (C_6_H_10_O_5_)n [4]. It is produced by various microorganisms, including fungi, bacteria, algae, and animals such as tunicates [5]. Cellulose exhibits several desirable characteristics, including low cost, renewability, sustainability, biodegradability, biocompatibility, and non-toxicity [6].

Nanocellulose (NC), cellulose with at least one dimension in the 1–100 nm range, offers high stiffness, low density, and a hydroxyl-rich surface, making it suitable for chemical functionalization. These qualities enable usage in packaging, composites, coatings, and separation media [7,8]. Broad adoption needs sustainable, efficient, scalable production methods, prompting process benchmarking [8,9,10,11,12,13,14].

Pineapple peel is an abundant agro-industrial residue in tropical countries and is a lignocellulosic biomass rich in cellulose, hemicellulose, and minor lignin. It has previously been reported to contain approximately 15–25% cellulose, making it a suitable substrate to produce nanocellulose via chemical routes and as a carbon source in fermentation-based systems [10,15,16,17,18,19,20,21,22]. Studies have demonstrated the extraction of nanocrystalline cellulose (CNC), nanofibrillated cellulose (NFC), and bacterial nanocellulose (BNC) from pineapple peel, either directly or via fermentation feed streams [21,23], and from other pineapple residues (e.g., crown fibers) [24]. Here, pineapple peel was used as the feedstock for the chemical hydrolysis route (HNC). At the same time, BNC was produced in a defined medium to provide a controlled biosynthesis reference for process-level benchmarking.

NC has been obtained through multiple methods, including chemical acid hydrolysis, mechanical disintegration, enzymatic treatment, and microbial fermentation (bacterial nanocellulose, BNC) [9,16,17]. NC’s performance, properties, and size depend on the extraction method, which involves factors such as instrumentation, equipment, input, and process conditions [18]. NC can be classified into three groups: cellulose nanocrystals (CNC), cellulose nanofibers (NFC), and BNC [14]. Lignocellulosic biomass (LCB) refers to plant biomass, which can be derived from wood and non-wood sources, such as agricultural residues and industrial wastes [15]. Using non-wood biomass presents an alternative for valorizing waste and contributes to the circular economy and sustainability [25]. On the other hand, BNC is secreted extracellularly by certain bacteria and has attracted increasing attention [1]. Compared to BNC, lignocellulosic biomass contains, in addition to cellulose, the main components of hemicellulose and lignin [15]. Because BNC and plant-derived NC originate from fundamentally different formation mechanisms and impurity profiles, they should be treated as distinct material classes with different manufacturing logic and downstream requirements.

One of the most common methods for NC production is the acid hydrolysis of agro-industrial wastes such as sugarcane bagasse, pineapple, wood, sawdust, and coffee husks [10,19,26]. These materials contain crystalline and amorphous regions of cellulose, with the amorphous areas easily hydrolyzed and the crystalline regions remaining intact [20]. This process produces cellulose nanocrystals or microfibrils, typically using sulfuric acid under controlled conditions to remove lignocellulosic material [19,21]. However, this involves multi-step chemical handling, extensive washing, and hazardous effluents, adding to process complexity and sustainability challenges.

On the other hand, BNC is secreted extracellularly as a network of polysaccharides, forming nano- and micrometer-sized fibers [16]. It is primarily produced by Gram-negative bacteria, such as Acetobacter, Azotobacter, Rhizobium, Agrobacterium, Pseudomonas, Salmonella, and Alcaligenes [9,16,22,23], as well as by Gram-positive bacteria, including *Sarcina ventriculi*, *Rhodococcus*, and *Lactobacillus hilgardii* [27]. Bacterial nanocellulose (BNC) and nanocellulose (NC) extracted from lignocellulosic biomass share the same molecular composition, consisting of β-(1→4)-linked D-glucose units; however, their structural organization and morphology differ significantly depending on the production method. BNC typically exhibits high crystallinity (~90%), flexibility, and excellent mechanical properties; moreover, it is essentially pure cellulose—free of lignin, hemicellulose, and pectin [23,28,29]. Additionally, its extraction and purification processes are simpler than those required for plant-derived cellulose [1]. At the same time, microbial biosynthesis is intrinsically carried out in aqueous nutrient media. It requires a stable producer strain, so its resource profile is governed by volumetric cultivation constraints rather than feedstock delignification and mineral-acid neutralization [30]. This reinforces the need to compare routes using explicitly defined, process-specific indicators rather than assuming one-to-one equivalence in inputs or outputs.

The genus Rhizobia includes Gram-negative soil bacteria that form symbiotic relationships with plants, creating nodules and fixing atmospheric nitrogen (N_2_) into ammonia (NH_3_) [31,32]. *Rhizobium leguminosarum* biovar *trifolii* is an agriculturally essential species in Uruguay, living saprophytically and in symbiosis with plant hosts [33]. Rhizobium leguminosarum biovar trifolii, an agriculturally important species, has been studied for its ability to produce bacterial nanocellulose (BNC) [34,35,36,37]. In this work, R. leguminosarum biovar trifolii served as a controlled BNC producer under static conditions. To compare two production methods, a decision matrix was developed that considered reagent use, processing time, yields, downstream processing, purification, and waste management.

This study addresses a process question: how do a top-down hydrolysis route and a bottom-up biosynthesis route compare when evaluated through the same sustainability-oriented criteria under controlled laboratory conditions? The aim is to map route-dependent trade-offs (e.g., chemical hazard and purification intensity versus cultivation time and volumetric constraints).

Despite significant advances in chemical and microbial methods, most comparative studies are limited to literature reviews, single-route optimization reports, or sustainability assessments that lack direct laboratory comparisons using a standard reporting format. In this work, we provide an experimentally based side-by-side benchmark comparing (i) sulfuric-acid hydrolysis of pineapple peel to produce hydrolyzed nanocellulose (HNC) and (ii) bacterial nanocellulose (BNC) biosynthesis by Rhizobium leguminosarum biovar trifolii in defined media. The novelty of this approach is the integration of standardized structural techniques (SEM, FTIR, AFM) with a clear multicriteria evaluation framework and a quantitative summary of process KPIs—including processing time, purification steps, downstream washing/neutralization water use, reagent hazard level, and waste classification—allowing for explicit comparison of sustainability and operational trade-offs across different routes [10,12,13,23].

## 2. Materials and Methods

### 2.1. Materials

Pineapple (*Ananas comosus*) peel residues were supplied by Congelados y Jugos del Valle Verde, located in Pital de San Carlos, Alajuela, Costa Rica. Sodium hydroxide (NaOH), sulfuric acid (H_2_SO_4_), hydrochloric acid (HCl), sodium hypochlorite (NaClO), and ethanol were purchased from Sigma-Aldrich (Steinheim, Germany). Pineapple peel was used exclusively as the lignocellulosic feedstock for the chemical hydrolysis route described in Section 2.2. *Rhizobium leguminosarum* biovar trifolii was kindly provided by Calister S.A. to the Laboratorio de Técnicas Nucleares Aplicadas a Bioquímica y Biotecnología, Centro de Investigaciones Nucleares-Facultad de Ciencias, Universidad de la República Uruguay, Montevideo, Uruguay. Bacterial nanocellulose (BNC) was produced using a defined culture medium (Section 2.3). All experiments were conducted in triplicate (*n* = 3). For AFM measurements, 30 cross-sections were analyzed for HNC and 25 for BNC.

### 2.2. Extraction and Purification of Hydrolyzed Nanocellulose (HNC) by Chemical Method

The procedure described by Camacho et al. [21] was followed with modifications to obtain hydrolyzed nanocellulose (HNC) from pineapple peel residues.

The pineapple peel residues were thoroughly washed with distilled water to remove adhering soluble sugars and residual fruit material, then dried in an oven at 50 °C for 24 h to standardize the moisture content and improve the reproducibility of the solid-to-liquid ratios. The dried biomass was then subjected to alkaline pretreatment using a 20 wt.% NaOH solution at 80 °C for 1.5 h under continuous agitation (300 rpm) to solubilize hemicelluloses/pectins, saponify extractives, and partially delignify the biomass. The material was washed with distilled water until it reached neutral pH, then filtered to stop the alkali reaction and remove dissolved impurities. A second alkali treatment was performed using a 12 wt.% NaOH solution at 70 °C for 1 h to remove residual non-cellulosic components further while limiting cellulose degradation, followed by neutralization with distilled water and filtration.

The resulting material was bleached with a 2.5 wt.% NaClO solution at 60 °C for 2 h under magnetic stirring to oxidatively remove residual lignin chromophores and improve cellulose purity. The product was subsequently treated with 17 wt.% HCl at room temperature for 2 h to neutralize residual alkaline species and remove inorganic salts/metal ions that can interfere with acid hydrolysis. The product was washed and filtered under vacuum using a BOECO filter (Hamburg, Germany, grade 389, pore size 5–8 µm) to separate the solid fraction from soluble by-products and standardize downstream handling.

Acid hydrolysis was carried out with 65 wt.% H_2_SO_4_ at 55 °C for 45 min under constant stirring (400 rpm) to preferentially hydrolyze amorphous cellulose domains and generate nanostructured cellulose while retaining more ordered domains. The hydrolyzed suspension was centrifuged at 4500 rpm for 10 min and washed repeatedly with distilled water until the pH reached neutrality to remove free acid and soluble reaction by-products and to minimize residual ionic species before characterization. Neutralization was confirmed by a pH meter (Hanna Instruments HI2211, Smithfield, RI, USA).

**Figure 1:**
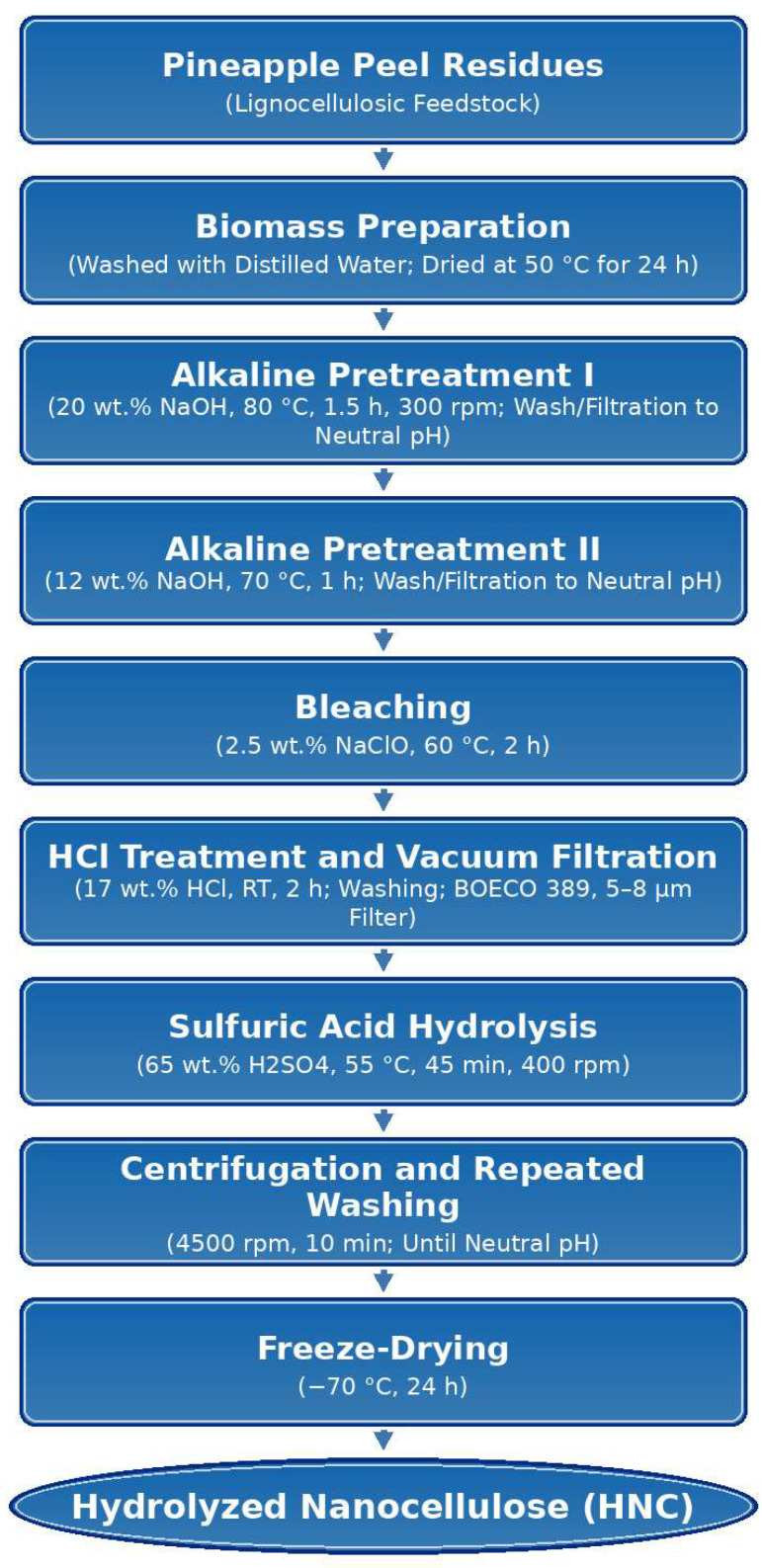
Diagram of the methodology for obtaining HNC from pineapple agroindustrial waste using the chemical method. Adapted from Camacho et al. [21].

The final nanocellulose suspension was freeze-dried at −70 °C for 24 h (Labconco FreeZone, Kansas City, MO, USA) to obtain a dry material while minimizing thermal degradation and preserving nanoscale features for subsequent characterization. Figure 1 illustrates the workflow of the chemical route for obtaining HNC from pineapple peel residues.

### 2.3. Production of Nanocellulose from Bacterial Cultures of Rhizobium leguminosarum Biovar Trifolii

The selection of *Rhizobium leguminosarum* biovar *trifolii* was based on its documented ability to synthesize extracellular cellulose, its agricultural relevance, and its availability from a certified microbial culture collection. Although less commonly studied than *Komagataeibacter* strains, *R. leguminosarum* has been successfully applied in previous reports of BNC production [38].

#### 2.3.1. Preparation of Bacterial Inocula

A loopful of 10-day-old colonies of *R. leguminosarum* biovar *trifolii* grown on yeast extract mannitol agar (YMA; 1 g/L yeast extract, 10 g/L mannitol, 0.5 g/L K_2_HPO_4_, 0.2 g/L MgSO_4_·7H_2_O, 0.1 g/L NaCl; pH 6.8) was transferred into 25 mL of yeast extract mannitol (YM) liquid medium. The initial inoculum was adjusted to an optical density (OD600) of approximately 0.1. Both YM and lactose-substituted YLL media were adjusted to pH 6.0 ± 0.2 before inoculation. Cultures were incubated statically (no agitation) at 30 °C in the dark for 3 days. Inoculum quality and consistency were verified by measuring the optical density (OD600) and using phase-contrast microscopy.

#### 2.3.2. BNC Production

One milliliter of the prepared inoculum (OD600 ≈ 0.1) was transferred into 100 mL of either YM or YLL medium contained in 250 mL Erlenmeyer flasks. The YLL medium consisted of 1 g/L yeast extract, 10 g/L lactose, 0.5 g/L K_2_HPO_4_, 0.2 g/L MgSO_4_·7H_2_O, and 0.1 g/L NaCl, with the pH adjusted to 6.0. All cultures were incubated statically at 30 °C in the dark for 15 days, relying on passive oxygen diffusion. Fermentation was carried out without mechanical agitation. At the end of cultivation, thick cellulose pellicles were recovered from the culture medium surface. Static cultivation was selected because pellicle formation is favored at the air–liquid interface, where oxygen availability is highest; shaking can alter oxygen transfer and introduce shear, potentially altering BNC morphology and complicating membrane harvesting. Ultrasonication was applied only after purification to disperse the purified network for AFM/SEM analysis and to reduce fiber bundling.

#### 2.3.3. Harvesting and Purification of the BNC

The BNC pellicles were harvested by vacuum filtration using 5–8 µm pore-size membrane filters and rinsed thoroughly with distilled water to remove residual culture medium.

**Figure 2:**
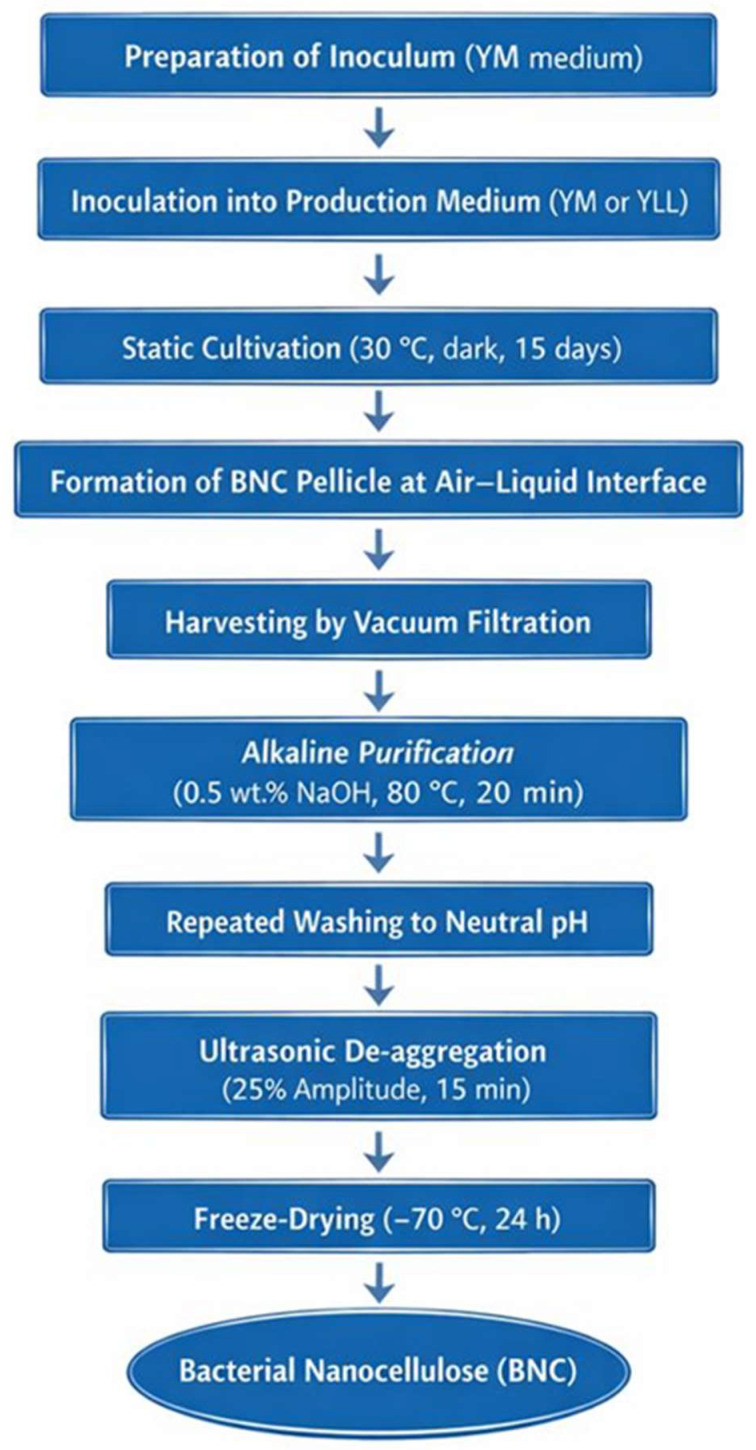
Workflow diagram of bacterial nanocellulose (BNC) production by *Rhizobium leguminosarum* biovar *trifolii* using defined culture media. The process includes inoculum preparation in yeast extract–mannitol (YM) medium, static cultivation in YM or YLL production medium, pellicle formation at the air–liquid interface, harvesting, alkaline purification, ultrasonic de-aggregation, and freeze-drying.

The membranes were then treated with 0.5% (*w*/*v*) NaOH at 80 °C for 20 min under agitation to eliminate bacterial cells and impurities. After alkaline treatment, the samples were filtered again and washed repeatedly with distilled water until the filtrate reached neutrality (pH 7.0 ± 0.2). The purified BNC pellicles were suspended in water and mechanically de-aggregated by ultrasonication using a titanium probe (6 mm diameter, 25% amplitude, 50 s on/10 s off cycles, 15 min total) to disrupt the arrangement of micro- and nanometric fibers [22,28]. The final suspension was stored at −70 °C for 24 h and subsequently freeze-dried (Labconco FreeZone, Kansas City, MO, USA). The lyophilized BNC samples were used for subsequent characterization. Figure 2 illustrates the overall workflow of BNC production by *R. leguminosarum* biovar *trifolii*.

### 2.4. Decision Matrix for the Comparison of NC Production Methods

The comparison of NC production methods was conducted by considering engineering and production aspects, with the evaluation criteria systematically organized into a decision matrix [38]. The scoring rubric used to assign performance levels (1–5) to each criterion is summarized in Table 1. Each criterion was assigned an importance value based on results from laboratory experiments. Importance levels ranged from 1 (lowest) to 5 (highest), and the importance value was multiplied by the corresponding criterion weight. The resulting products were then summed to yield a total score that supports a structured benchmarking of process burdens and trade-offs. The requirements and weights in the decision matrix were determined by combining published methodologies for process evaluation [38], established engineering practice, and consultation with process experts. Chemical hydrolysis and bacterial biosynthesis were chosen because they are the most widely adopted and industrially relevant routes for NC production from lignocellulosic biomass, as extensively documented in the literature [10,15,22,28].

Although a complete Life Cycle Assessment (LCA) and Techno-Economic Analysis (TEA) were not performed in this laboratory-scale study, the comparative framework was designed to be compatible with future LCA/TEA implementation. Specifically, we (i) define explicit system boundaries (distinguishing downstream washing/neutralization water from intrinsic fermentation medium volume), (ii) report inventory-relevant inputs and outputs (reagents, wash water, processing time proxies, and waste hazard class), and (iii) provide route-specific yield metrics with transparent normalization bases. These elements enable future ISO-aligned LCA and TEA once mass/energy balances and scale-dependent costs are available.

To confirm the robustness of the comparative outcome, a sensitivity analysis was performed, varying the weights and scores for each criterion within reasonable ranges. The overall ranking between chemical hydrolysis and bacterial biosynthesis remained unchanged, indicating that the decision matrix is stable and that moderate changes do not unduly influence the conclusions in the weighting scheme. The matrix is intentionally restricted to environmental and operational indicators (e.g., reagent burden, downstream washing/neutralization demand, purification complexity, and waste hazard class). It does not assess product equivalence, market substitutability, or application-specific performance. System boundary clarification:

“Water footprint” refers to the measured water used for post-synthesis washing/neutralization and downstream purification steps per batch. For the bacterial route, the culture volume is treated as a process medium input, whereas the additional rinse water used during purification is explicitly accounted for, thereby avoiding conflating cultivation volume with downstream washing demand. For transparency, the culture volume is still reported in Section 2.3.2 so that readers can reframe the boundary if desired.

The decision matrix presented in this section compares environmental and process-level indicators only (e.g., reagent use, purification steps, water footprint). It does not evaluate molecular or application-specific properties of the final nanocellulose materials. This framework is intended to benchmark early-stage sustainability indicators under controlled laboratory conditions.

Table 1 details the importance levels assigned to each criterion, based on their impact on overall efficiency, sustainability, and safety of the NC production process. Each criterion was evaluated according to key factors influencing production outcomes, and these have been quantified to reflect their relative significance:

- Amount of Reagents Used: Lower amounts (x ≤ 1 g) are highly valued (level 5) for cost and sustainability, while higher amounts (15 ≤ x) receive the lowest importance (level 1).
- Production/Acquisition Time: Shorter times (~3 days) are most valued (level 5); longer times (4 weeks) are least valued (level 1).
- Amount of Product Obtained: Higher yields (5 g or more) are most important (level 5); lower yields (5 mg) are least important (level 1).
- Water Footprint: Minimal water usage (x ≤ 3 L) is highly desirable (level 5); higher usage (15 L or more) is less desirable (level 1).
- Purification Stages: Fewer stages (1) are preferred (level 5); more stages (5) are less preferred (level 1).
- Waste Management: Non-hazardous waste is optimal (level 5); very hazardous waste is least favorable (level 1).

Additionally, quantitative indicators were collected throughout the experimental workflow to enable direct comparison of the two nanocellulose production methods. These included estimated process duration, the number of purification steps, the volume of water used, the type and quantity of reagents, and a qualitative assessment of post-treatment requirements. Data were recorded during the preparation, execution, and cleanup stages of both protocols (see Section 2.2 and Section 2.3). These values were compiled to complement the decision matrix, allowing for a more detailed assessment of operational and environmental metrics.

Beyond the numerical matrix, a radar chart was developed to visualize the comparative performance of each method across six key criteria: reagent usage, process duration, product amount, water footprint, purification complexity, and waste management. Each criterion was assigned a normalized score, ranging from 1 (least favorable) to 5 (most favorable), based on experimental data and process observations. These scores were plotted using Python’s Matplotlib library v3.10.8 to provide a holistic, intuitive visual comparison of each method’s strengths and weaknesses. This visualization is intended to summarize trade-offs across route-specific process burdens, not to claim direct comparability of substrate bases or product substitution.

### 2.5. Nanocellulose Characterization

#### 2.5.1. Fourier Transform Infrared Spectroscopy

Fourier transform infrared (FTIR) spectra were recorded on a Nicolet 6700 spectrophotometer (Thermo Scientific, Waltham, MA, USA) at LANOTEC CENAT, Pavas, Costa Rica, to identify functional groups in both hydrolyzed nanocellulose (HNC) and bacterial nanocellulose (BNC). Samples were analyzed over 4000–500 cm^−1^ with a spectral resolution of 4 cm^−1^, averaging 32 scans per spectrum. Measurements were performed in duplicate for each sample, using dried powders directly placed on the ATR (attenuated total reflectance) crystal. The ATR diamond crystal was cleaned with ethanol and dried between runs to avoid cross-contamination. Data were processed with OMNIC 8.1 software (Thermo Fisher Scientific).

#### 2.5.2. Scanning Electron Microscopy

The morphology of hydrolyzed nanocellulose (HNC) and bacterial nanocellulose (BNC) was examined using a JSM-6390LV scanning electron microscope (JEOL, Tokyo, Japan) at LANOTEC CENAT, Pavas, Costa Rica. Samples were coated with a thin gold layer (~10 nm) using a sputter coater (Quorum Q150R ES) to minimize charging effects. Observations were performed at an acceleration voltage of 20 kV, with a spot size of 40, under high-vacuum mode. Images were acquired at magnifications ranging from 100× to 10,000× to capture both micro- and nanostructural features. At least three independent fields were imaged per sample (*n* = 3), and all micrographs were recorded with internal scale bars for dimensional calibration.

#### 2.5.3. Atomic Force Microscopy

Atomic force microscopy (AFM) was performed using an Asylum Research MFP-3D system (Oxford Instruments, USA) at LANOTEC CENAT, operated in tapping mode to analyze the nanoscale morphology of hydrolyzed nanocellulose (HNC) and bacterial nanocellulose (BNC). A drop of each aqueous suspension was deposited onto freshly cleaved mica substrates and air-dried for 24 h under ambient conditions. Imaging was conducted using silicon nitride probes with a nominal tip radius of ~10 nm and a cantilever length of 100 μm. Typical scan areas ranged from 1 × 1 μm^2^ to 5 × 5 μm^2^, with a scan rate of 1 Hz. At least 30 cross-sections were measured for HNC and 25 for BNC (*n* = 3 independent replicates). The resulting topographic data were analyzed with WSXM software (version 4.0, Nanotec Electronica, Tres Cantos, Spain), and all measurements were calibrated against standard step-height gratings to ensure accuracy.

#### 2.5.4. Zeta Potential (ζ)

A dynamic light scattering instrument (N SZ-100V2, Horiba, Kyoto, Japan) measured zeta potential. Nanoparticle solutions containing a concentration of 0.1 mg/mL were analyzed.

#### 2.5.5. Data Analysis

The data were plotted in OriginPro 2019b (Northampton, MA, USA).

## 3. Results and Discussion

### 3.1. Structural and Physicochemical Characterization

#### 3.1.1. Fourier Transform Infrared Spectroscopy

**Figure 3:**
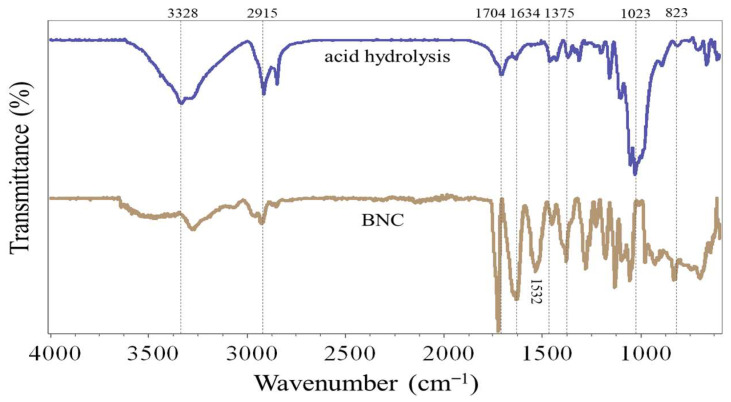
FTIR spectra of freeze-dried nanocellulose from acid hydrolysis (HNC) and bacterial biosynthesis (BNC).

FTIR analysis was used to identify the primary functional groups in freeze-dried nanocellulose samples obtained by acid hydrolysis (HNC) and bacterial biosynthesis (BNC) (Figure 3) [39]. Both spectra exhibited the characteristic absorption bands of cellulose, confirming the preservation of the polysaccharide backbone in both production routes. The broad band between 3000 and 3800 cm^−1^ corresponds to O–H stretching vibrations, typically linked to intramolecular and intermolecular hydrogen bonding as well as absorbed water [40,41,42]. The peak at 2900 cm^−1^ is associated with C–H stretching of aliphatic groups [43,44]. The band at 1030–1050 cm^−1^ arises from C–O–C stretching of glycosidic bonds in the polysaccharide backbone [41,42], while the absorption near 890 cm^−1^ is assigned to β-(1,4)-glycosidic linkages characteristic of cellulose [21,44,45].

Notable differences between HNC and BNC were observed in the region between 1704 and 1375 cm^−1^. In HNC, a distinct band at ~1704 cm^−1^ was detected, corresponding to C=O stretching vibrations. This signal is generally attributed to residual hemicellulose, acetyl groups, or carboxyl groups that were not entirely removed during pretreatment and hydrolysis [40,41,42]. Conversely, the BNC spectrum displayed only a weak or negligible band at ~1704 cm^−1^, whereas HNC showed a much stronger signal. This indicates higher overall purity in BNC, consistent with published bacterial cellulose spectra [22,28]. In addition, the region between 1375 and 1460 cm^−1^, assigned to C–H bending and CH_2_ wagging, showed differences in intensity and sharpness between the two samples. The sharper, more defined bands in BNC suggest a higher degree of crystallinity and structural order compared to HNC [43,44,46]. This result aligns with reports by Jacek et al. (2021) and Khan et al. (2021), which indicate that bacterial cellulose exhibits stronger CH_2_ wagging signals, indicative of ordered lattice structures [28,47].

Overall, the FTIR spectra confirm that both methods yield nanocellulose with the expected cellulose fingerprint. However, the reduced C=O signal and more defined CH_2_ bands in BNC emphasize greater chemical cleanliness and structural regularity under the present purification conditions, relative to HNC. These differences are interpreted as route-dependent outcomes and not as proof of universal superiority or industrial replacement.

#### 3.1.2. Scanning Electron Microscopy

**Figure 4:**
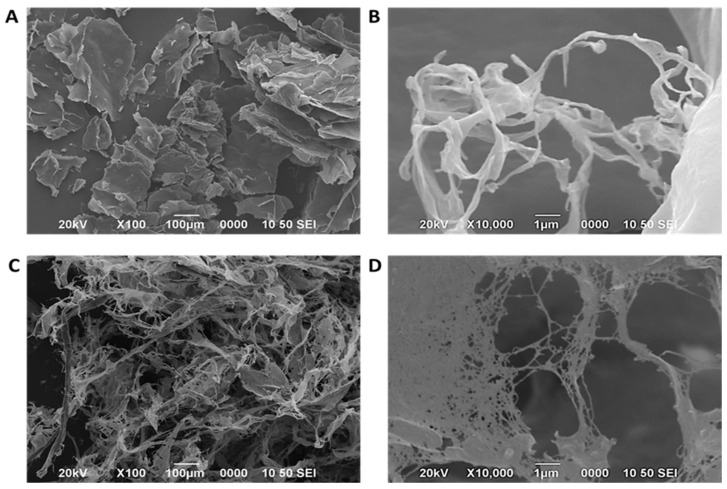
Scanning electron micrographs of nanocellulose obtained via acid hydrolysis (HNC) at (**A**) 100× and (**B**) 10,000× magnification, and via bacterial biosynthesis by *R. leguminosarum* at (**C**) 100× and (**D**) 10,000× magnification. Micrographs were acquired at an accelerating voltage of 20 kV.

The morphology of hydrolyzed nanocellulose (HNC) and bacterial nanocellulose (BNC) was investigated by SEM (Figure 4). All images were recorded with calibrated internal scale bars (100 μm for overview images and 500 nm for higher-magnification images).

The SEM micrographs of HNC revealed a lamellar-fibrous morphology (Figure 4A,B). Fibers were observed within the lamellae with widths typically ranging between 60 and 120 nm. This structural arrangement reflects the partial breakdown of cellulose fibrils by acid hydrolysis, which tends to produce heterogeneous aggregates. These findings are consistent with previous studies on acid-hydrolyzed pineapple and bagasse cellulose, which reported the presence of lamellar fragments accompanied by nanoscale fibrils [21,48].

In contrast, the BNC produced by *R. leguminosarum* formed a highly interconnected three-dimensional nanofiber mesh (Figure 4C,D). The fibrils displayed more homogeneous diameters, typically in the 30–60 nm range, which aligns with reports for bacterial cellulose obtained from *Komagataeibacter* species [28,47]. Network-like morphology is characteristic of extracellularly synthesized BNC, in which cellulose chains are secreted and self-assemble into ribbon-like nanofibers. The slight variability in fiber thickness can be attributed to the composition of the culture medium and the ultrasonic treatment applied during purification [22,49].

Overall, SEM observations confirm that HNC exhibits a more fragmented and heterogeneous morphology, while BNC demonstrates a finer, continuous, and homogeneous nanofiber network. These contrasts reflect different formation mechanisms (top-down cleavage vs. bottom-up biosynthesis) and are reported here descriptively, without implying that the two materials are interchangeable.

#### 3.1.3. Atomic Force Microscopy

**Figure 5:**
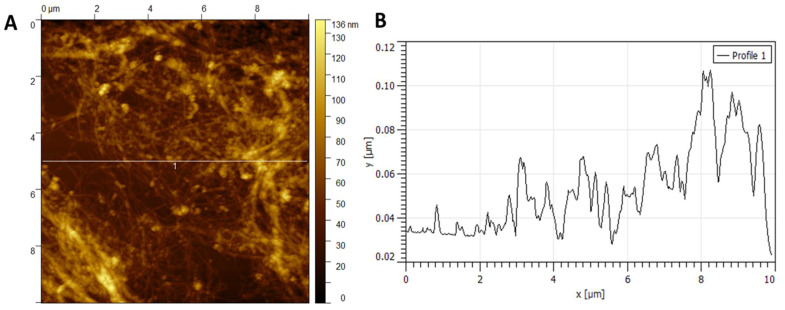
AFM of hydrolyzed nanocellulose (HNC): (**A**) surface morphology (X and Y axes in μm); (**B**) height cross-section profile (Z axis in nm) taken along the horizontal line indicated in panel (**A**). The horizontal line and the numerical marker are software-generated reference indicators used to extract the height profile and do not represent normalized or scaled values. Average fibril height = 70 ± 12 nm (mean ± 95% confidence interval).

**Figure 6:**
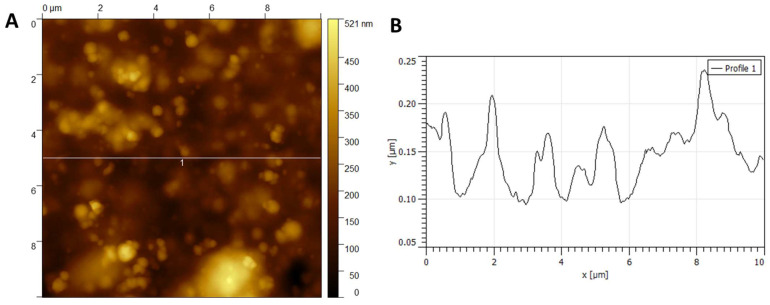
AFM of bacterial nanocellulose (BNC): (**A**) surface morphology (X and Y axes in μm); (**B**) height cross-section profile (Z axis in nm) taken along the horizontal line indicated in panel (**A**). The horizontal line and the numerical marker correspond to instrumental reference indicators generated by the AFM software and have no independent physical meaning beyond profile extraction. Average fibril height = 37 ± 9 nm (mean ± 95% confidence interval).

Figure 5 and Figure 6 show the AFM images of NC obtained by chemical hydrolysis (HNC) and by *Rhizobium leguminosarum* (BNC), respectively. In Figure 5A, dispersed fibrils can be observed in the range of 20–60 nm. Figure 5B presents the cross-section profile.

In AFM images (Figure 5 and Figure 6), the X and Y axes correspond to the scanned surface dimensions (in micrometers), while the Z axis represents the topographic height (in nanometers). Average height values were obtained from multiple independent cross-sections in different sample regions (*n* = 30 for HNC and *n* = 25 for BNC). Although individual profiles may vary (e.g., 40–100 nm), the statistical analysis of these measurements yielded mean heights of 70 ± 12 nm for HNC (Figure 5) and 37 ± 9 nm for BNC (Figure 6). These values are reported with 95% confidence intervals, providing a statistically robust description of the variability observed across measurements.

In Figure 6A, the nanocellulose obtained by the bacterial method (BNC) exhibits a uniform network of nanofibrils. In contrast, the chemically hydrolyzed nanocellulose (HNC) shows a more heterogeneous, lamellar morphology (Figure 4). Figure 6B corresponds to a cross-section of Figure 6A, confirming that the BNC fibrils are thinner and more homogeneously distributed compared with the lamellar structures observed in HNC. These differences reflect the distinct mechanisms of nanocellulose formation: bottom-up extracellular biosynthesis in BNC versus top-down acid hydrolysis of lignocellulosic structures in HNC.

The differences in nanofibril dimensions can be attributed to the distinct mechanisms of formation and purification. BNC is biosynthesized extracellularly as a highly crystalline network of thin fibers by *R. leguminosarum*. At the same time, acid hydrolysis relies on top-down cleavage of plant cell wall structures, producing broader and more heterogeneous fibrils [22,28]. Moreover, the ultrasonic disaggregation applied during BNC purification further reduces fiber bundling and enhances dispersion, contributing to the finer diameter observed [49].

#### 3.1.4. Comparative Summary of Physicochemical Properties

Table 2 summarizes the physicochemical differences between HNC and BNC, integrating the results from FTIR, SEM, and AFM. Both materials preserved the fundamental cellulose backbone, as indicated by the characteristic FTIR bands (O–H stretching, C–H stretching, and β-(1,4)-glycosidic linkages). However, HNC exhibited a residual C=O band at ~1704 cm^−1^, reflecting the presence of acetyl or hemicellulosic residues. In contrast, this band was absent from BNC, confirming lower residual non-cellulosic signatures under the applied purification protocol, consistent with microbial cellulose literature [22,28].

Morphologically, SEM revealed that HNC formed a lamellar-fibrous structure with fibrils in the range of 60–120 nm, consistent with prior reports of acid-hydrolyzed cellulose from pineapple and sugarcane residues [21,48]. Conversely, BNC displayed a homogeneous three-dimensional nanofiber mesh, with fibril diameters ranging from 30 to 60 nm, in agreement with bacterial cellulose reported for *Komagataeibacter* strains [28,47].

AFM measurements further confirmed these distinctions: HNC fibrils averaged 70 ± 12 nm in height, while BNC fibrils averaged 37 ± 9 nm. These values, validated by 95% confidence intervals, emphasize the more uniform and thinner morphology of BNC compared to the broader, heterogeneous fibrils of HNC. Similar ranges for BNC have been reported in the literature (30–50 nm), confirming the reproducibility of the bacterial biosynthetic pathway [28].

Compared with previous studies, the novelty of this work lies not in asserting equivalence between HNC and BNC, but in benchmarking their production processes under controlled laboratory conditions. While acid hydrolysis of pineapple waste has been reported [21], and bacterial cellulose production has been extensively studied with Komagataeibacter [22,28,47], few works have juxtaposed these two routes with respect to both structural properties and process-level indicators (e.g., water footprint, waste, and purification steps).

In summary, the comparative analysis demonstrates that BNC consistently exhibits higher chemical cleanliness under the applied washing conditions, narrower fibril size distribution, and superior structural homogeneity. In contrast, HNC is more heterogeneous and retains minor chemical residues. These findings describe route-dependent differences and do not imply that one product replaces the other across applications.

### 3.2. Visual Integration of Multicriteria Evaluation

Table 3 presents the values obtained for each criterion, enabling comparison of the two processes used to obtain NC. Based on the decision matrix, the bacterial method scored 66 points, compared with 51 for acid hydrolysis. This numerical difference reflects the selected criteria and weighting under the defined boundary (environmental/operational burden). It should be interpreted as a scenario-specific benchmarking output rather than an absolute “winner” between non-equivalent industrial products. In contrast to chemical processes that use toxic reagents and consume large amounts of washing/neutralization water, biological methods often exhibit advantages in selected sustainability indicators under laboratory conditions [8,30,50,51].

#### 3.2.1. Amount of Reagents Used

This evaluation criterion was determined by the amount of reagents consumed during each process. According to the chemical method description, it uses up to 4 reagents. In contrast, the bacterial method uses multiple reagents in the culture media during both the maintenance and inducer phases. These sum to 6 reagents per phase. However, the quantity of reagents used in the bacterial process is significantly lower, measured in grams. Because reagent “count” alone does not capture concentration, hazard class, or neutralization demand, this criterion is interpreted here as a pragmatic proxy for reagent burden within the selected scoring framework, not as a claim of direct chemical comparability between routes. A possible alternative to reduce the amount of reagents used and lower production costs is to explore alternative culture media derived from residual biomass. In recent years, several studies have been conducted to produce BNC using various waste products, such as apple peels, cantaloupe juice, and *Ulva lactuca*, as well as reducing sugar from grass straw, grass husk, wheat husk, and corn cob [28,30,44,50,51].

#### 3.2.2. Production Time

Regarding the time required to obtain NC, there is a notable difference between the chemical and bacterial methods. The chemical method requires constant monitoring throughout its execution, as each stage demands control of temperature, agitation, and washes [15,21].

The chemical process typically takes about 1 week and involves multiple stages requiring active monitoring, including alkaline pretreatment, bleaching, acid hydrolysis, and neutralization [21]. In contrast, the bacterial method spans approximately 18 days, which includes inoculum preparation and 15 days of static fermentation. While this route requires minimal supervision once inoculated, the total calendar time is notably longer than that of the chemical pathway.

Additionally, the extraction and purification of membranes and fibers produced during fermentation take approximately 2.5 h and require minimal intervention. Accordingly, “time” can be interpreted either as calendar time or as hands-on operational effort; this study reports both qualitatively to avoid misleading equivalence.

#### 3.2.3. Water Footprint

The water impact of both methods was considered due to their significant water consumption. In the chemical method, an estimated 14 L of water is used for the neutralization stage with NaOH. In contrast, the bacterial process uses only 300 mL to remove the same reagent. Here, “water footprint” is reported as the measured downstream washing/neutralization water required to reach neutral pH and remove process chemicals after product formation. Separately, the bacterial route necessarily operates in an aqueous culture medium (100 mL per flask in this study), which is an intrinsic process medium rather than post-synthesis wash water; therefore, the 0.3 L value should not be interpreted as the total water involved in fermentation. Thus, the amount of water consumed during the chemical method’s washing stages could support the establishment of multiple fermentation cultures. For example, 14 L would correspond to 28 cultures, each prepared on a pilot scale with 500 mL.

Most chemical methods still need pre-treatment, acid hydrolysis, bleaching, and neutralization, which consume large quantities of water and toxic chemical reagents [8,15,52]. Due to this issue, many researchers have investigated the use of microorganisms to produce BNC [30,51,53,54] as an alternative that may meet the demand for this material while maintaining process sustainability and preserving the physicochemical characteristics of the NC. It has been reported that BNC production decreases water consumption [29]. Because reported water metrics are boundary-dependent (wash water vs. total process water including culture medium), we explicitly specify the boundary above to prevent misinterpretation.

#### 3.2.4. Purification Stages

There is a significant difference in the number of purification stages between the methods. In chemical extraction, all residual reagents from the raw material must be removed. An alkali treatment was applied to remove components such as lignin, hemicelluloses, pectins, and waxes, and a bleaching process was used to remove lignin residues. Additionally, the final product may require extensive washing under constant stirring to remove sulfuric acid and soluble materials, such as salts and sugars, until neutralization is achieved. However, it is still necessary to develop efficient pretreatments to remove impurities, contaminants, and non-cellulosic components on the raw materials without affecting cellulose or its purity, as cellulose is the primary material used for NC preparation [8,15,55].

In bacterial production, the purification stage is built into the process, as the bacterial route inherently yields high-purity cellulose, making purification straightforward and cost-effective. Moreover, although BNC shares the same cellulose chemistry as plant-derived NC, it lacks the other plant components (no lignin, hemicellulose, or pectin) [23,28,29,51].

In practical terms, the bacterial route still requires post-cultivation removal of cell and medium residues (e.g., alkaline treatment and washing). Still, it avoids delignification/bleaching and strong-mineral-acid removal steps that dominate the downstream burden of plant-biomass hydrolysis.

#### 3.2.5. Waste Management

Reagents used in acid hydrolysis are considered corrosive and toxic, particularly at high concentrations, thereby increasing their risk [8,15,56]. In contrast, reagents used in bacterial biosynthesis pose lower risks due to significantly lower concentrations and the possibility of waste-derived culture media [30,51]. Their disposal involves sterilization and either regular waste disposal or effluent treatment. On the other hand, acid hydrolysis requires chemical reagents at various stages, including NaOH, NaClO, HCl, and H_2_SO_4_ [21,23]. This methodology requires neutralizing acidic and basic waste before disposal, thereby increasing water and reagent consumption [8,15]. Therefore, the chemical method is hindered by the use of toxic chemicals, high energy consumption, prolonged time consumption, and wastewater generation [8,15,56]. In addition, this process also leads to equipment corrosion. It poses challenges in disposing of and recovering the large amounts of chemical waste generated [8,15] and in complying with local, regional, national, or international regulations (C(2001)107/FINAL of the OECD). Therefore, developing green technologies for NC production is being prioritized.

#### 3.2.6. Quantitative Process Comparison

To complement the scoring-based decision matrix in Table 3, Table 4 provides a direct quantitative comparison of key process parameters for the two nanocellulose production methods, HNC and BNC. This comparison highlights differences in operational complexity, environmental impact, and resource intensity, enriching the assessment with numerical and structural data.

Chemical hydrolysis, although relatively fast (~7 days), involves multiple highly controlled steps, including alkaline pretreatment, bleaching, acid hydrolysis, and neutralization. These steps require intensive manual oversight and consume large volumes of water—approximately 14 L per batch—primarily for washing and pH adjustment. The method relies on several concentrated chemical reagents (e.g., NaOH, HCl, H_2_SO_4_), which increase handling risks and generate hazardous waste that must be neutralized and disposed of in accordance with environmental regulations. Equipment, corrosion, and operational safety concerns are additional limitations of this approach.

In contrast, bacterial biosynthesis using *Rhizobium leguminosarum* biovar *trifolii* occurs over a more extended period (~15–18 days) but requires minimal intervention once the cultures are inoculated. Water usage is significantly lower, with only ~300 mL needed per batch to rinse and purify the resulting BNC membranes. However, the bacterial route inherently operates in an aqueous culture medium (0.1 L per flask here), which constitutes an additional water-associated process input distinct from downstream rinse water. The reagents are non-toxic and are used at low concentrations in culture media, thereby reducing costs and environmental burden. Additionally, the purification process is more straightforward and inherently less hazardous, since bacterial NC is secreted extracellularly in high purity without requiring intensive chemical treatment.

Morphologically, NC products also differ. HNC presents a more fragmented, lamellar structure with thicker fibrils (average height: 70 ± 12 nm), while bacterial NC displays a finer, interconnected nanofiber network with thinner fibrils (average height: ~37 nm), as observed in AFM and SEM analyses. These structural differences may influence surface area, mechanical behavior, and application potential in biomedical engineering or biocomposites.

In addition to these process parameters, yields were quantified in this study: HNC reached 3.15% (*w*/*w*) relative to dry biomass, and BNC reached 1.065 g/L in the culture medium. These measured values define the productivity–sustainability trade-off observed in our experiments. For context, the literature reports HNC yields from pineapple peel of 0.12–0.15 g/g of dry biomass, depending on pretreatment intensity and hydrolysis conditions [10,21,23]. These literature values (≈12–15% *w*/*w*) are higher than the 3.15% (*w*/*w*) yield observed here, reflecting differences in pretreatment severity, solid-to-liquid ratios, hydrolysis conditions, and yield normalization approaches. We therefore report yields transparently as measured under our standardized laboratory protocol and avoid direct yield-based “winner” claims across non-equivalent production bases (dry biomass vs. culture volume).

**Figure 7:**
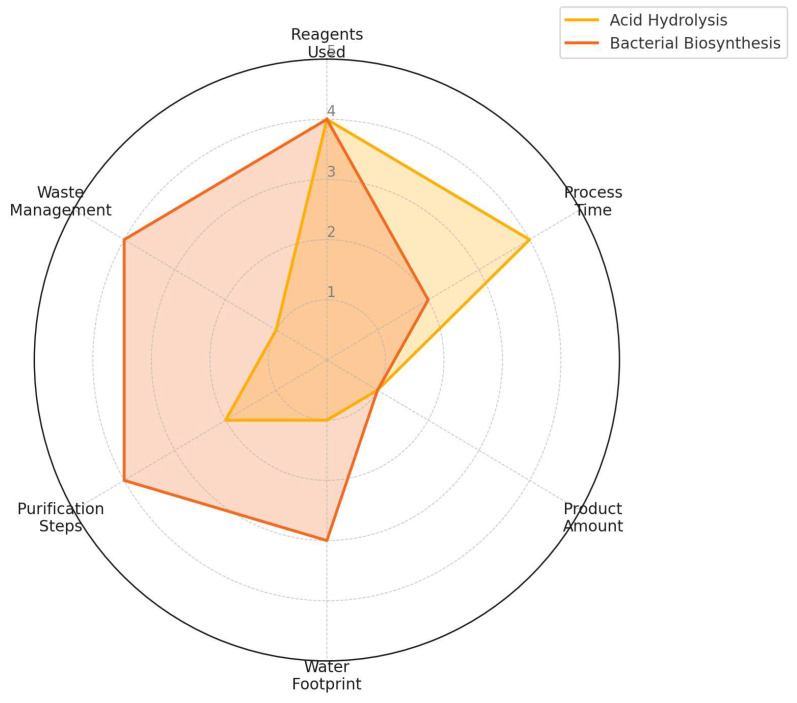
Radar chart comparing the normalized scores of six criteria for nanocellulose production by acid hydrolysis (HNC) and bacterial biosynthesis (BNC). The radial scale ranges from 1 (least favorable) to 5 (most favorable), corresponding to the scoring levels defined in the decision matrix (Table 1). Lower values indicate greater process burden, while higher values indicate better environmental and operational performance within the defined system boundary.

To complement the numerical evaluation in Table 3, Figure 7 presents a radar chart that visually compares the weighted scores assigned to each decision criterion for NC production between the acid hydrolysis and bacterial biosynthesis methods.

The six criteria included are (1) amount of reagents used, (2) production/acquisition time, (3) amount of product obtained, (4) water footprint, (5) purification steps, and (6) waste management. These scores were derived from experimental observations and normalized using a consistent scale from 1 (least favorable) to 5 (most favorable), based on environmental and operational performance.

The chart illustrates route-dependent trade-offs: bacterial biosynthesis scores higher in downstream washing demand, purification simplicity, and waste safety, while acid hydrolysis performs better in calendar time. This visualization is intended to summarize trade-offs under the chosen criteria and boundary, not to imply industrial substitutability or a universal best route.

Previous studies have reported yields for nanocellulose production from pineapple waste ranging from several tens to a few hundred milligrams per gram of dry substrate, depending on the process and conditions used [10,21,24]. Large-scale production remains challenging, and values exceeding 10 g/L are rare, except in highly optimized or specialized systems. These data collectively support the novelty and competitiveness of the dual-route approach presented here.

#### 3.2.7. Surface Charge and Colloidal Stability

Zeta potential measurements were performed to evaluate the surface charge and colloidal stability of both nanocellulose suspensions. The HNC samples exhibited a ζ-potential of −41.0 mV, while the BNC samples showed slightly less negative values, averaging −38.5 ± 1.0 mV. Both results indicate strong electrostatic repulsion (|ζ| > 30 mV), confirming the stability of the colloidal dispersions.

The more negative ζ-potential of HNC is attributed to sulfate ester groups formed during sulfuric acid hydrolysis, which increase the surface charge density. Conversely, the less negative potential in BNC reflects hydroxyl-terminated surfaces free of sulfate substitution, consistent with its biosynthetic origin.

## 4. Limitations and Future Work

While this study benchmarks a pineapple-peel-based chemical hydrolysis route (HNC) against a microbial biosynthesis route (BNC) performed in a defined medium, several limitations must be acknowledged from a process-comparability standpoint. First, all experiments were conducted at a laboratory scale, which may not fully reflect the dynamics, challenges, or costs of industrial-scale production. In addition, because the two routes do not share a common starting biomass in the present work, the comparative interpretation is intentionally limited to process-level indicators (e.g., downstream purification burden, reagent hazard, and washing/neutralization demand) rather than substrate-equivalent conversion performance.

A further limitation is that the bacterial route did not employ pineapple-derived hydrolysates. This choice ensured reproducibility; however, future work will adapt the culture medium to agro-industrial effluents or pineapple enzymatic hydrolysates and will explicitly establish a shared starting basis (e.g., pineapple-derived sugars or hydrolysates) to enable strictly valid, mass-balanced comparisons between routes. Parameters such as energy consumption, bioreactor performance, and downstream logistics were not assessed, yet they are critical for real-world deployment.

Additionally, yield quantification, particularly in terms of dry mass per liter or per gram of biomass, was not the primary focus of this work. Although both routes produced sufficient material for characterization, future studies should include precise yield analysis under varying substrate concentrations, fermentation conditions, and scaling factors. Importantly, future experiments should report yields using a harmonized functional unit (e.g., grams of nanocellulose per kilogram of dry pineapple peel input, and/or per kilogram of fermentable sugar derived from pineapple), so that productivity is evaluated on a standard accounting basis rather than using mixed normalizations. The use of fed-batch or continuous culture systems may further improve productivity in the bacterial route.

Moreover, although the decision matrix integrated environmental and process-related criteria, no life-cycle assessment (LCA) or techno-economic analysis (TEA) was conducted. These tools are necessary to validate the long-term sustainability and market feasibility of each pathway. Incorporating full LCA/TEA into future work would enable policymakers and industry stakeholders to make informed decisions on integrating green materials. Such evaluations should also define clear system boundaries (e.g., distinguishing process wash/neutralization water from intrinsic fermentation medium volume) to avoid boundary-driven misinterpretation of water-related indicators.

This study did not include direct mechanical characterization (e.g., tensile strength, modulus, flexibility) of the NC samples due to limitations in sample yield and the availability of laboratory instrumentation. However, previous work has reported that both chemically and bacterially derived nanocelluloses can achieve high mechanical performance, with tensile strengths typically ranging from 100 to 300 MPa and moduli of up to several GPa, depending on the processing route and post-treatment [1,6,16]. As our research advances to pilot scale, future studies will focus on systematically evaluating the mechanical properties and application-specific performance of NC produced from pineapple waste and on defining performance–process trade-offs without implying product substitutability.

Genetic engineering of *R. leguminosarum* and optimization of metabolic pathways may also enable higher cellulose production rates, allowing this species to approach the productivity levels reported for conventional high-producing strains such as *Komagataeibacter xylinus*, while maintaining controllable cultivation conditions. Similarly, exploring low-cost nutrient sources, such as sugar-rich agro-industrial effluents or enzymatically treated biomass, including pineapple-derived sugar streams, could further reduce input costs and environmental burdens.

Finally, a broader assessment that includes NC functionality—such as rheology, crystallinity, thermal stability, or surface charge—would complement this comparative framework, enabling application-driven selection of the most suitable production method. In parallel, future work should expand the benchmarking framework to include robust mass/energy balances and standardized reporting formats that make route comparisons transparent and reproducible.

Overall, future research should focus on the convergence of environmental impact, economic feasibility, and advanced material performance to facilitate the adoption of nanocellulose technologies in next-generation circular bioeconomies.

## 5. Conclusions

This study presented a side-by-side benchmarking of two nanocellulose production routes under controlled laboratory conditions: hydrolyzed nanocellulose (HNC) obtained by sulfuric acid hydrolysis of pineapple peel waste, and bacterial nanocellulose (BNC) produced by *Rhizobium leguminosarum* biovar *trifolii* in a defined medium. The benchmarking integrates structural characterization (SEM, FTIR, AFM) with sustainability-oriented process indicators, including reagent burden, production time, downstream washing/neutralization water, purification steps, and waste management.

Quantitative analyses confirmed structural and physicochemical differences consistent with the distinct formation mechanisms. HNC exhibited fibril heights of 70 ± 12 nm with a heterogeneous lamellar-fibrous morphology, whereas BNC displayed thinner fibrils averaging 37 ± 9 nm and a more homogeneous three-dimensional nanofiber mesh. FTIR spectra showed that HNC retained a residual C=O band at ~1704 cm^−1^, indicative of remaining non-cellulosic signatures (e.g., hemicellulose/acetyl groups), while BNC presented only a weak to negligible residual signal in this region, consistent with higher chemical cleanliness under the applied purification scheme.

Additionally, the experimental productivity and surface charge of both materials were determined. Hydrolyzed nanocellulose (HNC) yielded 3.15% (*w*/*w*) relative to dry pineapple peel, while bacterial nanocellulose (BNC) reached 1.065 g/L in the culture medium. Because these values use different normalization bases (dry biomass vs. culture volume), they are not directly comparable. They are reported only to provide a transparent representation of route-specific productivity metrics. Both suspensions exhibited colloidal stability (|ζ| > 30 mV). Although HNC had a higher ionic charge due to sulfate esterification, BNC showed greater uniformity, lower residual non-cellulosic signatures, and lower downstream chemical hazard.

The unique contribution of this work lies in benchmarking a top-down chemical hydrolysis pathway against a bottom-up bacterial biosynthesis pathway by integrating structural characterization (SEM, FTIR, AFM) with sustainability-oriented process indicators, including water usage, purification complexity, waste management, and operational burden. Importantly, the reported “water footprint” values refer to downstream washing/neutralization demand (~14 L for HNC vs. ~0.3 L rinse water for BNC), while acknowledging that bacterial biosynthesis inherently occurs in an aqueous culture medium, which represents an additional process input. This boundary clarification is essential to avoid over-interpreting water-use comparisons across fundamentally different process architectures.

In conclusion, although bacterial biosynthesis requires longer cultivation time (~18 days versus ~7 days for chemical hydrolysis) and operates under volumetric cultivation constraints, it offers clear advantages in downstream purification simplicity, reduced hazardous effluents, and structural homogeneity. Conversely, chemical hydrolysis enables direct valorization of lignocellulosic pineapple residues with a faster cycle time but higher reagent hazard and a greater downstream washing/neutralization burden. Both routes, therefore, represent non-equivalent yet complementary strategies, and the present benchmarking framework is intended to support process decision-making rather than to declare a universal “winner.” Future work should connect both routes through a shared pineapple-derived carbon basis (e.g., pineapple hydrolysates/sugars) and apply harmonized functional units to enable strictly mass-balanced, substrate-equivalent comparisons.

## Figures and Tables

**Table 1:** Scoring rubric (1–5) for decision-matrix criteria.

Criteria			Score Level		
	1 (Least Favorable)	2	3	4	5 (Most Favorable)
Number of reagents used	x ≥ 15	9 ≤ x ≤ 14	5 ≤ x ≤ 8	2 ≤ x ≤ 4	x ≤ 1
Production/acquisition time	4 weeks	3 weeks	2 weeks	1 week	~3 days
Amount of product obtained	x ≤ 5 mg	5 < x ≤ 10 mg	0.01 < x ≤ 0.5 g	0.5 < x ≤ 1 g	x ≥ 5 g
Water footprint	x ≥ 15 L	10 ≤ x ≤ 14 L	7 ≤ x ≤ 9 L	4 ≤ x ≤ 6 L	x ≤ 3 L
Purification stages	5	4	3	2	1
Waste management	Very hazardous	Hazardous	Requires caution	Low hazard	Non-hazardous

Note: x denotes the measured value per batch within the defined experimental system boundary. For “Number of reagents used” and “Amount of product obtained”, x is expressed in grams (g). For “Water footprint”, x is expressed in liters (L) of downstream washing/neutralization water per batch.

**Table 2:** Summary of physicochemical characterization of nanocellulose obtained from both methods.

Method	FTIR Key Bands (cm^−1^)	SEM Morphology	AFM Average Height (nm)	Structure Type	Interpretation of Chemical Cleanliness
HNC	890, 1030–1050, 2900	Lamellar + Fibrous	70 ± 12	Dispersed Fibers	Plant-derived; residual carbonyl signature detected (~1704 cm^−1^)
BNC	890, 1030–1050, 2900	3D Nanofiber Mesh	37 ± 9 *	Interconnected Network	Microbial origin; weaker carbonyl signature under applied purification

* AFM height for BNC is based on *n* = 25 measured cross-sections; average and 95% confidence interval are reported in the text (Section 3.1.3). Product purity is inferred from process pathway and FTIR analysis (Section 3.1.1). Interpretation is based on FTIR features (Section 3.1.1) and process pathway, and is not a claim of universal product superiority.

**Table 3:** Decision matrix with the values obtained for each criterion to compare the acid hydrolysis and bacterial biosynthesis methods.

Criteria	Method *				
	Criterion Weight (w)	Acid Hydrolysis	Total Acid Hydrolysis	Bacterial Biosynthesis	Total Bacterial Biosynthesis
Amount of used reagents	3	4	12	4	12
Production/Acquisition time	5	4	20	2	10
Amount of product obtained	4	1	4	1	4
Water footprint	4	1	4	3	12
Purification	4	2	8	4	16
Waste management	3	1	3	4	12
Total	51			66	

* Note: This matrix benchmarks environmental and operational indicators under the defined system boundary. Route-specific yield metrics are reported separately as 3.15% (*w*/*w*, dry biomass basis) for HNC and 1.065 g/L (culture volume basis) for BNC, and are not used to claim substrate-equivalent productivity across routes.

**Table 4:** Quantitative comparison of both nanocellulose production methods.

Criterion	HNC	BNC
Total processing time	~7 days (intensive monitoring)	~18 days (minimal monitoring)
Amount of reagents	High (NaOH, HCl, H_2_SO_4_, etc.)	Low (medium components)
Estimated water usage	~14 L	~0.3 L
Culture medium volume (reported separately)	Not applicable	0.1 L per flask (this study)
Waste toxicity	High	Low
Purification steps	~5	~2
Fiber size (AFM)	70 ± 12 nm	37 ± 9 nm
Zeta potential (ζ)	−41.0 mV	−38.5 ± 1.0 mV

## Data Availability

The original contributions presented in this study are included in the article. Further inquiries can be directed to the corresponding author.
